# Maternal Downward Neighborhood Income Mobility and Ensuing Severe Neonatal Morbidity

**DOI:** 10.1001/jamapediatrics.2024.6667

**Published:** 2025-02-17

**Authors:** Jennifer A. Jairam, Eyal Cohen, Christina Diong, Howard Berger, Hilary K. Brown, Jun Guan, Joel G. Ray

**Affiliations:** 1 Department of Medicine Research, St Michael’s Hospital, Toronto, Ontario, Canada; 2 Child Health Evaluative Sciences, SickKids Research Institute, Toronto, Ontario, Canada; 3 ICES, Toronto, Ontario, Canada; 4 Department of Medicine, St Michael’s Hospital, University of Toronto, Toronto, Ontario, Canada; 5 Department of Health and Society, University of Toronto Scarborough, Scarborough, Ontario, Canada

## Abstract

This population-based cohort study examines changes in mothers’ neighborhood income between 2 consecutive births and associated morbidity/mortality outcomes in their newborns.

Severe neonatal morbidity (SNM) is an indicator of neonatal well-being and quality of perinatal care^1,2^ and is more frequent following a severe complication during birth or in the neonatal period.^3^

Cross-sectional data suggest that newborns of mothers residing in socioeconomically deprived neighborhoods are at higher risk of morbidity.^4^ However, no study to date has evaluated outcomes among newborns following a notable decrease in maternal neighborhood income between 2 pregnancies, a potentially important period when preventive interventions might be offered to mothers and newborns at risk. This study assessed the association of SNM and neonatal mortality (SNM-M) with a change in the mother’s neighborhood income between 2 births.

## Methods

This population-based cohort study used linked administrative health data from Ontario, Canada, from April 2002 to March 2022 within a universal health care system (eTable 1 in Supplement 1). Included were all women with at least 2 consecutive singleton hospital-based births at 20 to 42 weeks’ gestation residing within neighborhood income quintiles 2 to 5 at the time of the first birth, with income quintile 5 being the highest (eTable 2 in Supplement 1). Quintile 1 residents were excluded, as their upward mobility was studied previously.^4^ The use of deidentified data in this project was authorized under section 45 of Ontario’s Personal Health Information Protection Act and exempt from a research ethics board review and informed consent. This study followed the STROBE reporting guideline.

The main exposure was degree of maternal neighborhood income mobility between the first and second births, creating 7 mutually exclusive exposure groups: moved down by 1, 2, or 3 or more quintiles or moved up by 1, 2, or 3 quintiles, each relative to no change in neighborhood income quintile (referent), between births. The primary outcome (model A) was a validated composite of SNM-M at the second birth, using the Canadian Neonatal Adverse Outcomes Indicator (eTable 2 in Supplement 1).^3^ The secondary study outcome (model B) was SNM-M or stillbirth.

Modified Poisson regression generated relative risks (RRs) and 95% CIs.^5^ RRs were adjusted for maternal and newborn characteristics. Details of all study variables and analyses are reported in eTable 2 and eMethods in Supplement 1.

## Results

Among the 575 616 mothers included (mean [SD] age, 32.1 [4.6] years), 140 475 (24.4%) experienced downward neighborhood income mobility, 118 742 (20.6%) upward neighborhood income mobility, and 316 399 (55.0%) no neighborhood income mobility (Table). Women with downward neighborhood income mobility had the highest prevalence of multiple antecedent comorbidities (8.7%) and younger age at the second birth (Table).

**Table.** Mother and Newborn Characteristics by Maternal Neighborhood Income Quintile (Q) Mobility

Characteristic	Neighborhood income mobility between 2 births, No. (%)		
	Any downward (n = 140 475)	Any upward (n = 118 742)	None (n = 316 399)
Neighborhood income Q of mother at first birth			
Q2	22 639 (16.1)^a^	53 822 (45.3)^b^	71 798 (22.7)
Q3	33 219 (23.6)	40 595 (34.2)^b^	79 038 (25.0)
Q4	42 515 (30.3)	24 325 (20.5)^b^	88 015 (27.8)
Q5 (Highest)	42 102 (30.0)^a^	NA	77 548 (24.5)
No. of maternal comorbidities 1-365 d before second birth			
0-2^c^	128 303 (91.3)^a^	111 422 (93.8)	299 857 (94.8)
≥3^c^	12 172 (8.7)^a^	7320 (6.2)	16 542 (5.2)
Mother at second birth			
Age, mean (SD), y	31.2 (5.1)^a^	32.4 (4.5)	32.4 (4.5)
Months between first and second births, median (IQR)	36 (26-54)^a^	36 (26-52)^b^	29 (22-39)
Live birth parity 1	106 240 (75.6)	92 336 (77.8)	246 180 (77.8)
Live birth parity ≥2	34 235 (24.4)	26 406 (22.2)	70 219 (22.2)
Rural residence	16 682 (11.9)	13 584 (11.4)	31 009 (9.8)
Nonrefugee immigrant	30 397 (21.6)	24 606 (20.7)	62 393 (19.7)
Newborn at first birth			
SNM-M or stillbirth	9627 (6.9)	7811 (6.6)	22 081 (7.0)
Newborn at second birth			
Stillbirth	497 (0.4)	346 (0.3)	930 (0.3)
Female sex	68 209 (48.6)	57 623 (48.5)	153 578 (48.5)
Birth weight, median (IQR), g	3427 (3100-3756)	3450 (3133-3770)	3450 (3140-3771)
Gestational age at birth, median (IQR), wk	39 (38-40)	39 (38-40)	39 (38-40)
Preterm live birth <32 wk gestation	857 (0.6)	568 (0.5)	1681 (0.5)
Preterm live birth <37 wk gestation	7854 (5.6)	5773 (4.9)	15 727 (5.0)
Any congenital or chromosomal anomaly	4405 (3.1)	3653 (3.1)	9859 (3.1)

Abbreviation: NA, not applicable.

^a^ Represents a standardized difference >0.10 comparing mothers with downward mobility with mothers without income mobility.

^b^ Represents a standardized difference >0.10 comparing mothers with upward mobility with mothers without income mobility.

^c^ Total number of Johns Hopkins Adjusted Clinical Group System Aggregated Diagnosis Groups (excluding pregnancy diagnosis), 1 to 365 days before the second birth hospitalization.

Relative to that for newborns of mothers with stationary neighborhood income between births, the adjusted RR for SNM-M at the second birth was 1.08 (95% CI, 1.04-1.13) for newborns of mothers with downward neighborhood income movement by 2 quintiles and 1.14 (95% CI, 1.07-1.20) for those of mothers with downward movement by 3 or more quintiles (Figure). In contrast, a lower associated risk of SNM-M was observed by degree of upward neighborhood income quintile mobility (Figure, A). A similar pattern was seen for SNM-M or stillbirth (Figure, B).

**Figure.**
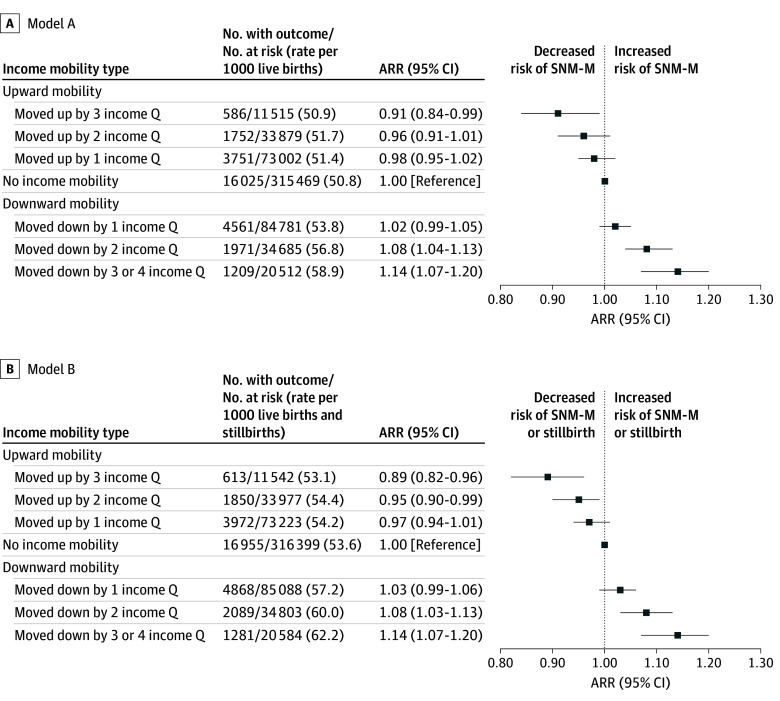
Maternal Neighborhood Income Quintile (Q) Mobility and Severe Neonatal Morbidity or Neonatal Mortality (SNM-M) and Stillbirth Risk Degree of maternal neighborhood income Q mobility between 2 consecutive births and risk of (A) SNM-M and (B) SNM-M or stillbirth, each assessed at the second birth. Relative risks are adjusted for neighborhood income Q at the first birth, SNM-M or stillbirth at first birth, maternal age at second birth, birth interval, parity, number of comorbidities within 1 to 365 days before second birth, residence at second birth, and immigrant status. Model A is also adjusted for any newborn congenital or chromosomal anomaly identified within the second birth hospitalization. ARR indicates adjusted relative risk.

## Discussion

In this study, downward neighborhood income movement between births was associated with a higher risk of SNM-M. The opposite was observed for upward neighborhood income mobility. As a limitation, a mother’s individual-level income loss or gain may not be congruent with her neighborhood income mobility, and vice versa. We lacked information on maternal individual-level income, race, and adverse life events preceding and following the first birth.

Mothers residing in the lowest income neighborhood quintile were previously shown to experience less SNM-M if they had upward neighborhood income mobility between births.^4^ The underlying reasons for the prior^4^ and current findings are multifactorial and appreciated within a life-course model.^6^ Accordingly, research is needed to identify those factors that predispose a woman to downward neighborhood income mobility, especially after birth, to elucidate causal pathways and to investigate whether financial incentives or neighborhood improvements can reduce adverse pregnancy and neonatal outcomes.

## Notes

Supplement 1. **eTable 1.** List of ICES Databases Used in the Current Study **eTable 2.** Cohort Entry and Exclusion Criteria, Methods, and Coding to Identify Study Outcomes
eMethods

eReferences

Supplement 2.
Data Sharing Statement
