# Supplementary material for: Maternal Downward Neighborhood Income Mobility and Ensuing Severe Neonatal Morbidity
Source: JAMA Pediatr. 2025 Feb 17;179(4):463–5. doi: 10.1001/jamapediatrics.2024.6667 (PMC11833651; doi:10.1001/jamapediatrics.2024.6667)
Supplement: Supplement 1. — eTable 1. List of ICES Databases Used in the Current Study eTable 2. Cohort Entry and Exclusion Criteria, Methods, and Coding to Identify Study Outcomes eMethods eReferences [file jamapediatr-e246667-s001.pdf]

Supplemental Online Content

Jairam JA, Cohen E, Diong C, et al. Maternal downward neighborhood income mobility and ensuing severe neonatal morbidity. *JAMA Pediatr.* Published online February 17, 2025. doi:10.1001/jamapediatrics.2024.6667

eTable 1. List of ICES Databases Used in the Current Study

eTable 2. Cohort Entry and Exclusion Criteria, Methods, and Coding to Identify Study Outcomes

eMethods

eReferences

This supplemental material has been provided by the authors to give readers additional information about their work.

© 2025 Jairam JA et al. *JAMA Pediatrics*.

eTable 1: List of ICES Databases Used in the Current Study

**Dataset name**	**Description**
Aggregated Diagnosis Groups (ADG)	The Johns Hopkins Adjusted Clinical Groups (ACGs)® system assigns an ICD code to one of 32 diagnosis clusters known as Aggregated Diagnosis Groups (ADG). Individual diseases or conditions are placed into a single ADG based on 5 clinical dimensions: duration of the condition; severity of the condition; diagnostic certainty; etiology of the condition; and specialty care involvement. ICD codes within the same ADG are similar in both clinical criteria and expected need for healthcare resource. Individuals may have multiple diagnoses and belong to multiple ADGs (between zero and 32 ADGs).
Canadian Institute for Health Information Discharge Abstract Database (CIHI-DAD)	Captures all in-patient hospital admission records including obstetric deliveries and deaths. Diagnostic codes are based on the *International Statistical Classification of Diseases and Related Health Problems, Tenth Revision, Canada* (*ICD-10-CA*), and procedural codes are based on the *Canadian Classification of Health Interventions (CCI)*.
Immigration, Refugees and Citizenship Canada Permanent Residents Database (IRCC-PRD)	Captures demographic information on all international migrants who obtained permanent residency in Canada from January 1985 to March 2023.
Linked Delivering Mothers and Newborns (MOMBABY)	Derived from CIHI-DAD, provides linked inpatient hospital admission records of mothers and their infants.
National Ambulatory Care Reporting System (NACRS)	Collects data on hospital-and community-based ambulatory care, such as day surgery, outpatient and community-based clinics and emergency departments.
Ontario Health Insurance Plan Claims Database (OHIP)	Contains information about inpatient and ambulatory visits, consultations and procedures provided to Ontario residents eligible for Ontario's publicly funded health insurance system by fee-for-service health care practitioners (e.g., physicians, optometrists, laboratories for diagnostic tests etc.).
Postal Code Conversion File Plus (PCCF+)	A digital file that links the Canada Post Corporation (CPC) six-character postal code and Statistics Canada's standard geographic areas (e.g., dissemination area). Area-level income quintiles range from Q1 (lowest) to Q5 (highest) income neighbourhoods.
Registered Persons Database (RPDB)	Includes vital status and sociodemographic information about all individuals who have ever received an Ontario Health Insurance Plan (OHIP) number (e.g., date of birth, sex, and postal code).
Same Day Surgery (SDS)	Contain demographic, diagnostic, procedural and treatment information about all day surgical procedures.
Statistic Canada Census	Information from the Canadian Census, statistical information about the population including population

© 2025 Jairam JA et al. *JAMA Pediatrics*.

Dataset name	Description
Aggregated Diagnosis Groups (ADG)	The Johns Hopkins Adjusted Clinical Groups (ACGs)® system assigns an ICD code to one of 32 diagnosis clusters known as Aggregated Diagnosis Groups (ADG). Individual diseases or conditions are placed into a single ADG based on 5 clinical dimensions: duration of the condition; severity of the condition; diagnostic certainty; etiology of the condition; and specialty care involvement. ICD codes within the same ADG are similar in both clinical criteria and expected need for healthcare resource. Individuals may have multiple diagnoses and belong to multiple ADGs (between zero and 32 ADGs).
	counts and various levels of geography (e.g., census metropolitan areas, communities, census tracts etc.)

© 2025 Jairam JA et al. *JAMA Pediatrics*.

eTable 2: Cohort Entry and Exclusion Criteria, Methods, and Coding to Identify Study Outcomes

Assessment	Timing	Disease, procedure or condition	ICD-10-CA or CCI codes in CIHI-DAD, SDS and NACRS	Diagnostic & fee codes in OHIP	Other sources
Inclusion criteria	April 1, 2002 to March 31, 2022, at the time of the mother's index delivery hospitalization date, for the first and second births (selected) in the study cohort	• All hospital singleton livebirths and stillbirths at $20^{0/7}$ to $42^{0/7}$ weeks' gestation among women who were initially residing in an income quintile (Q) Q2, Q3, Q4, or Q5 area in Ontario at the time of their first birth during the study period, and who had a second consecutive birth also in Ontario.<br>• Among mothers with more than two births in the study period, an earlier livebirth or stillbirth was randomly selected to serve as the *first birth* in the cohort. A consecutive livebirth served as the *second birth*.<br>• Births limited to women aged 15 to 50 years old with a valid OHIP/IKN number from MOMBABY for the first and second index delivery hospitalizations.<br>• Stillbirth: fetal death arising *in utero*, or a newborn with no signs of life at birth, at ≥ 20 weeks' gestation -- among livebirths and stillbirths.	MOMBABY	--	RPDB
	Same as above	• Mother's postal code will be used to derive neighbourhood income Q.	--	--	RPDB, PCCF+, Statistic Canada census data
Exclusion criteria applied to all birth records	Same	• Records with warning for mother's IKN or KEY	MOMBABY/RPBD: warning for IKN/KEY (WARN not =”N”(No Warning)). Include N=no warning	--	--
	At the mother's index delivery hospitalization for the first birth	• Multiple births	MOMBABY (M_MULTIBIRTH=’T’ or B_MULTIBIRTH=’T’)	--	--
	At the infant's index birth hospitalization for the first birth	• Infant gestational age at birth is <20 or ≥43 weeks' gestation or missing	MOMBABY:<br>a. B_GESTWKS_DEL not in MOMBABY<br>b. gestational age <20 or ≥43 weeks' gestation	--	--
	At the mother's index delivery hospitalization for the first birth	• Women without a second consecutive birth during study period	MOMBABY	--	--

© 2025 Jairam JA et al. *JAMA Pediatrics*.

Assessment	Timing	Disease, procedure or condition	ICD-10-CA or CCI codes in CIHI-DAD, SDS and NACRS	Diagnostic & fee codes in OHIP	Other sources
	Same	• Women who had an invalid OHIP number or hospital number	MOMBABY/RPDB:<br>Invalid M_IKN (maternal IKN)<br>a. VALIKN ne 'V'<br>b. M_IKN not in RPDB (according to no sex and no bdate)<br>c. M_IKN with sex ='M' in RPDB	--	--
	At the time of arrival to Canada	• Immigrants with a landing date prior to their birth date	RPDB	--	IRCC-PRD
	Same	• Refugees and other immigrants	--	--	IRCC-PRD
Exclusion criteria applied to the first selected livebirth or stillbirth during the study period	At the mother's index delivery hospitalization for the first birth	Women aged < 15 or > 50 years, or age missing	MOMBABY/RPDB:<br>M_IKN age < 15 or > 50 or missing	--	--
	Same	Women who were a non-Ontario resident	--	--	RPDB:<br>M_IKN non-Ontario resident (substr (prcddablk, 1,2 ne '35')
	Same	Women ineligible for OHIP	--	--	RPDB
	Same	Women residing in income Q1 neighbourhoods or missing an income Q	--	--	RPDB, PCCF+, Statistics Canada census data
	Same	Rural/urban residence missing	--	--	RPDB, PCCF+
Exclusion criteria applied to the second selected livebirth during the study period	At the mother's index delivery hospitalization for the second birth	Women aged < 15 or > 50 years, or age missing	MOMBABY/RPDB:<br>M_IKN age < 15 or > 50 or missing	--	--
	Same	Women who were a non-Ontario resident	--	--	RPDB:<br>M_IKN non-Ontario resident

© 2025 Jairam JA et al. *JAMA Pediatrics*.

Assessment	Timing	Disease, procedure or condition	ICD-10-CA or CCI codes in CIHI-DAD, SDS and NACRS	Diagnostic & fee codes in OHIP	Other sources
	Same	Women missing an income Q	--	--	RPDB, PCCF+, Statistics Canada census data
	Same	Women ineligible for OHIP	--	--	RPDB
	270 days or less during the 365-day lookback period before the mother's hospitalization for the second birth	Eligible for OHIP	--	--	RPDB
	Same	Rural/urban residence missing	--	--	RPDB, PCCF+
	At the time of arrival to Canada & the mother's delivery hospitalization date for the second birth	Immigrants with a landing date after their admission date for the second birth	RPDB	--	IRCC-PRD
	At the time of arrival to Canada	Women recorded as a non-refugee immigrant and Canadian-born, or missing their country of birth	--	--	IRCC-PRD
	At the mother's index delivery hospitalization for the first and second births	< 161 days between these dates	MOMBABY: B_BDATE	--	--
	At the infant's birth hospitalization for the second birth	Live born infant birthweight < 250 g or missing	MOMBABY	--	--
	At the infant's birth hospitalization discharge date for the second birth	Live born infant missing their birth hospitalization discharge date	MOMBABY	--	--

© 2025 Jairam JA et al. *JAMA Pediatrics*.

Assessment	Timing	Disease, procedure or condition	ICD-10-CA or CCI codes in CIHI-DAD, SDS and NACRS	Diagnostic & fee codes in OHIP	Other sources
Main study exposure	At the mother's index delivery hospitalization for the first and second births	***Degree of downward neighbourhood income mobility*** and ***degree of upward neighbourhood income mobility*** between the first & second consecutive births vs ***no income mobility*****:**<br>i) Moved down one income Q<br>ii) Moved down two income Q<br>iii) Moved down three/four income Q<br>iv) Moved up one income Q<br>v) Move up two income Q<br>vi) Moved up three income Q<br>vii) No income mobility (referent): remaining in the same income Q area (i.e., Q2, 3, 4, or 5) between the first & second consecutive births	--	--	RPDB, PCCF+, Statistic Canada census data
Main outcome	If the birth hospitalization length of stay was ≤ 27 days, then SNM-M was further assessed within any subsequent rehospitalization, up to 27 days after birth.<br><br>If the birth hospital duration was > 27 days, then SNM-M was assessed any time during that hospitalization, even if it surpassed 27 days.	Severe neonatal morbidity (SNM) – among livebirths	Birth Trauma<br>P10.0 to P10.3, P13.0, P13.2, P13.3, P14.0, P14.1 P14.2, P14.3, P14.8, P14.9, P13.4<br><br>Necrotising enterocolitis<br>P77<br><br>Seizure<br>P90, R56<br><br>Intraventricular haemorrhage<br>P52.1, P52.2<br><br>Cerebral infarction<br>I63	--	Based on validated Canadian NAOI[a]

© 2025 Jairam JA et al. *JAMA Pediatrics*.

Assessment	Timing	Disease, procedure or condition	ICD-10-CA or CCI codes in CIHI-DAD, SDS and NACRS	Diagnostic & fee codes in OHIP	Other sources
			Periventricular leukomalacia<br>P91.2<br><br>Hypoxic ischaemic encephalopathy<br>P91.5, P91.6, P91.8<br><br>Infection<br>P36 G00-03, G05, A40, A41.5, A41.8, A41.9, B95.1, B96.2<br><br>Respiratory distress syndrome<br>P22.0<br><br>Chronic respiratory disease originating in the perinatal period<br>P27<br><br>Pneumonia<br>P23, J12-J18<br><br>Other respiratory<br>P28.0, P28.5<br><br>Perinatal intestinal perforation<br>P78.0<br><br>Retinopathy of prematurity<br>H35.1<br><br>Ventilatory support<br>1GZ31CAEP, 1GZ31CAND, 1GZ31CAPK, 1GZ31CBND, 1GZ31CRND, 1GZ31GPND, 1GZ31JAMD, 1GZ31JANC, 1GZ31JAPK		

© 2025 Jairam JA et al. *JAMA Pediatrics*.

Assessment	Timing	Disease, procedure or condition	ICD-10-CA or CCI codes in CIHI-DAD, SDS and NACRS	Diagnostic & fee codes in OHIP	Other sources
			Pneumothorax requiring intercostal catheter 1GV52DA, 1GV52DATS, 1GV52HA, 1GV52HAHE, 1GV52HATK, 1GV52LA, 1GV52LATS, 1GV52LAXXE, 1GV54JATS, 1GV55JATS, Any body cavity surgical procedure 1AA52, 1AA87, 1AC87, 1AE87, 1AF87, 1AG87, 1AJ87, 1AK87, 1AN52, 1AN59, 1AN87, 1AP59, 1AP72, 1AP87, 1AW59, 1AW72, 1AW87, 1AX87, 1BA72, 1BA80, 1BA87, 1BB72, 1BB80, 1BB87, 1BD72, 1BD80, 1BD87, 1BF80, 1BG72, 1BG80, 1BG87, 1BK59, 1BM72, 1BM80, 1BM87, 1BN72, 1BN80, 1BN87, 1BP72, 1BP80, 1BP87, 1BQ72, 1BQ80, 1BQ87, 1BS72, 1BS80, 1BS87, 1BT72, 1BT80, 1BT87, 1GA87, 1GA89, 1GB87, 1GB89, 1GD89, 1GE80, 1GE87, 1GE89, 1GE91, 1GH84, 1GJ86, 1GJ87, 1GK87, 1GK89, 1GM80, 1GM86, 1GM87, 1GN92, 1GR87, 1GR89, 1GR91, 1GT78, 1GT87, 1GT89, 1GT91, 1GV87, 1GV89, 1GW87, 1GX80, 1GX86, 1GX87, 1GY70, 1GY72, 1GY86, 1HJ76, 1HJ82, 1HN87, 1HP76, 1HP78, 1HP80, 1HP82, 1HP83, 1HP87, 1HR80, 1HR84, 1HR87, 1HS80 (excl. 1HS80G), 1HS90, 1HT80 (excl. 1HT80G), 1HT89, 1HT90, 1HU80 (excl. 1HU80G), 1HU90, 1HV80 (excl. 1HV80G), 1HV90, 1HW78, 1HW79, 1HX80, 1HX87, 1HX80, 1HZ87, 1IA76,		

© 2025 Jairam JA et al. *JAMA Pediatrics*.

Assessment	Timing	Disease, procedure or condition	ICD-10-CA or CCI codes in CIHI-DAD, SDS and NACRS	Diagnostic & fee codes in OHIP	Other sources
			1IA80, 1IA87, 1IB76, 1IB79, 1IB80, 1IB82, 1IB87, 1IC76, 1IC80, 1IC82, 1IC87, 1ID76, 1ID80, 1ID82, 1ID86, 1ID87, 1IF83, 1IJ76, 1IJ80, 1IM76, 1IM80, 1IM82, 1IM83, 1IM87, 1IN83, 1IN84, 1IN87, 1JE57 (excl. 1JE57G), 1JE76, 1JE80, 1JE87, 1JJ76, 1JJ80, 1JK76, 1JK80, 1JK87, 1JW51 (excL. 1JW51G), 1JW57, 1JW76, 1LA84, 1LC84, 1LD84, 1NA72, 1NA74, 1NA76, 1NA77, 1NA80, 1NA84, 1NA86, 1NA87, 1NA88, 1NA89, 1NA90, 1NA91, 1NA92, 1NE80, 1NF76, 1NF78, 1NF80, 1NF82, 1NF84, 1NF86, 1NF87 (excl. 1NF87B), 1NF89, 1NF90, 1NF91, 1NF92, 1NK76, 1NK77, 1NK80, 1NK82, 1NK84, 1NK87 (excl. 1NK87B), 1NM74, 1NM76, 1NM77, 1NM80, 1NM82, 1NM87 (excl. 1NM87B), 1NM89, 1NM91, 1NP72, 1NP73, 1NP86, 1NQ74 (excl. 1NQ74B), 1NQ80, 1NQ84, 1NQ86, 1NQ87 (excl. 1NQ87B), 1NQ89, 1NQ90, 1NT80, 1NT84, 1NT86, 1NT87, 1NV89, 1OA87, 1OB87, 1OB89, 1OD76, 1OD89, 1OE76, 1OE80, 1OE89, 1OJ76 (excl. 1OJ76B), 1OJ87, 1OJ89, 1OK87, 1OK89, 1OK91, 1OT72, 1OT87, 1OT91, 1PB87, 1PB89, 1PC80, 1PC87 (excl. 1PC87D), 1PC89, 1PC91, 1PE57 (excl. 1PE57BD), 1PE80 (excl. 1PE80D), 1PE82, 1PE87 (excl. 1PE87D), 1PE89 (excl. 1PE89D), 1PG76, 1PG77, 1PG80 (excl. 1PG80D), 1PG86, 1PG89, 1PL74 (excl. 1PL74CD), 1PL80, 1PM79, 1PM86, 1PM87 (excl. 1PM87B),		

© 2025 Jairam JA et al. *JAMA Pediatrics*.

Assessment	Timing	Disease, procedure or condition	ICD-10-CA or CCI codes in CIHI-DAD, SDS and NACRS	Diagnostic & fee codes in OHIP	Other sources
			1PM89, 1PM90, 1PM91, 1PM92, 1QE53, 1QE80, 1QE82, 1QE84, 1QE87, 1QE89, 1QG89, 1QM74, 1QM80, 1QM87, 1QM89, 1QM91, 1QN82, 1QT87, 1QT91, 1RB74, 1RB80, 1RB83, 1RB87, 1RB89,1RD89, 1RF51, 1RF72, 1RF74, 1RF80, 1RF87, 1RF89, 1RM87 (excl. 1RM87B), 1RM89, 1RM91, 1RN87, 1RN89, 1RS74, 1RS80, 1RS86, 1RS87, 1RS89, 1RW87, 1RW88, 1RW91, 1RW92, 1SA74, 1SA75, 1SA80, 1SA89, 1SC74, 1SC75, 1SC80, 1SC87, 1SC89, 1SE53, 1SE89 (excl. 1SE89D), 1SF80, 1SF87, 1SF89, 1SG80, 1SG87, 1SH87, 1SM74, 1SM80, 1SM87, 1SN87, 1SN93, 1SQ53, 1SQ74, 1SQ80, 1SQ87, 1SQ91, 1SQ93, 1SW74, 1SY80, 1SY84, 1SY87, 1SZ87, 1VA53, 1VA74, 1VA75, 1VA80, 1VA87, 1VA93, 1VC74, 1VC80, 1VC87, 1VC91, 1VC93, 1VE80, 1VG53, 1VG55, 1VG72, 1VG73, 1VG74, 1VG75, 1VG80, 1VG87, 1VG93, 1VK80, 1VK87, 1VK89, 1VL80, 1VL87, 1VM80, 1VM87, 1VN80, 1VN87, 1VP74, 1VP80, 1VP87, 1VP89, 1VQ74, 1VQ79, 1VQ80, 1VQ82, 1VQ87, 1VQ91, 1VQ93, 1VS72, 1VS80, 1VX87<br><br>Resuscitation by intubation and/or chest compressions<br>1.GZ.30^^ 1.HZ.30^^		

© 2025 Jairam JA et al. *JAMA Pediatrics*.

Assessment	Timing	Disease, procedure or condition	ICD-10-CA or CCI codes in CIHI-DAD, SDS and NACRS	Diagnostic & fee codes in OHIP	Other sources
			Central venous or arterial catheter insertion<br>1KV53HACH, 1KV53HAFT, 1KV53LAFT, 2IM28GP, 2LZ28GQPL, 2LZ28GRPL, 2LZ28JAPL, 1KX53HACH, 1KX53HAFT*, 1KX53LAFT*, 2LZ28GQPL, 2LZ28GRP, 1IS53^^<br>* Up until 2015 umbilical venous catheterization (UVC) was classified in these codes. After 2015, they were found in 1IS53^^.<br><br>Administration of any intravenous fluid<br>1.LZ.35^^<br><br>Transfusion of red blood cells or a blood product<br>1LZ19HHU1A, 1LZ19HHU1J, 1LZ19HHU2A, 1LZ19HHU2J, 1LZ19HHU3J, 1LZ19HHU4J, 1LZ19HHU5J, 1LZ19HHU6A, 1LZ19HHU6J, 1LZ19HHU9A, 1LZ19HHU9J, 1LZ19HMU1, 1LZ19HMU2, 1LZ19HMU9, 1LZ35HAC5<br>Note: CIHI has a "BTANY" variable which also summarizes all the above		
	Same	All-cause neonatal mortality – among livebirths	--	--	RPDB
Secondary outcome	Same	SNM-M (as defined above) or stillbirth (i.e., fetal death arising *in utero*, or a newborn with no signs of life at birth, each at ≥ 20 weeks' gestation)	MOMBABY (m_stillbirth)	--	--
Descriptive variables and covariates	At the mother's index delivery hospitalization for the first and second births	Neighbourhood income quintile (Q2, 3, 4 or 5) at the first birth<br><br>Neighbourhood income quintile (Q1, 2, 3, 4 or 5) at the second birth	--	--	RPDB, PCCF+, Statistic Canada census data

© 2025 Jairam JA et al. *JAMA Pediatrics*.

Assessment	Timing	Disease, procedure or condition	ICD-10-CA or CCI codes in CIHI-DAD, SDS and NACRS	Diagnostic & fee codes in OHIP	Other sources
	1 to 365 days prior to the hospitalization for the second birth	Number of comorbidities: Total number of Aggregated Diagnosis Groups (ADGs), excluding any pregnancy defined ADG.	ADGs are obtained from diagnosis codes in DAD, SDS and NACRS using The Johns Hopkins ACG® System Version 10 software	--	--
	At the mother's index delivery hospitalization for the second birth	Maternal age: 16-24, 25-29, 30-50 years	MOMBABY: M_AGE	--	--
	Same	Parity: number of previous livebirths (Previous term deliveries + Previous preterm deliveries), operationalized as a binary variable: 2 or more livebirths vs. 1 livebirth.	CIHI-DAD/MOMBABY	--	--
	Same	Rural vs. urban residence	--	--	RPDB, PCCF+
	At the time of arrival to Canada	Immigrant status: non-refugee immigrants vs. non-immigrants. (An immigrant woman must have been born outside of Canada and then migrated to, and obtained permanent residency, in Ontario).	--	--	IRCC-PRD
	The infant's birth admission dates, for first and second births	Interpregnancy birth interval: Time elapsed between infant's birth admission, for first and the second births (in months). Calculated by subtracting the infant's birth admission date for the first birth from the infant's birth admission date for the second birth. Categories: 6-17, 18-60, or ≥ 61 months	MOMBABY: B_BDATE	--	--
	At the infant's birth hospitalization for the first birth	SNM-M (as defined above) or stillbirth (i.e., fetal death arising *in utero*, or a newborn with no signs of life at birth, each at ≥ 20 weeks' gestation)	MOMBABY (m_stillbirth)	--	--
	At the infant's birth hospitalization for the second birth	Infant's biological sex assigned at birth	MOMBABY: b_sex	--	--
	Same	Birthweight: 250-1499, 1500-2499, 2500-3999, ≥ 4000 g	MOMBABY	--	--
	Same	Gestational age at birth, in weeks	MOMBABY	--	--
	Same	Preterm birth < 32 weeks' gestation – among livebirths	MOMBABY:B_GESTWKS_DEL	--	--

© 2025 Jairam JA et al. *JAMA Pediatrics*.

Assessment	Timing	Disease, procedure or condition	ICD-10-CA or CCI codes in CIHI-DAD, SDS and NACRS	Diagnostic & fee codes in OHIP	Other sources
	Same	Preterm birth < 37 weeks' gestation – among livebirths	MOMBABY:B_GESTWKS_DEL	--	--
	Between 0 days (at the infant's birth hospitalization [for the second birth]) & up to the hospitalization discharge date	Any congenital or chromosomal anomaly – among livebirths	ICD-10 Q00-Q99	--	--

[a] Nelson CRM, Ray JG, Auger N, Moore AM, Little J, Murphy PA, Van den Hof M, Shah PS; Canadian Perinatal Surveillance System, Public Health Agency of Canada. Neonatal Adverse Outcomes among Hospital Livebirths in Canada: A National Retrospective Study. Neonatology 2024 Aug 22:1-8. doi: 10.1159/000540559.

Abbreviations: ACG: Adjusted Clinical Group; ADG: Aggregated Diagnosis Group; CIHI: Canadian Institute for Health Information; DAD: Discharge Abstract Database; CCI: Canadian Classification of Health Interventions; ICD-10-CA: International Classification of Diseases, 10th Revision, Canada; IRCC-PRD: Immigration, Refugees, and Citizenship Canada Permanent Resident Database; NACRS: National Ambulatory Care Reporting System; OHIP: Ontario Health Insurance Plan; PCCF+: Postal code conversion file plus (Statistics Canada); RPDB: Registered Persons Database; SDS: Same Day Surgery Database.

© 2025 Jairam JA et al. *JAMA Pediatrics*.

eMethods

Data Sources

All study administrative datasets were linked using unique identifiers, held and analyzed at ICES, an independent, non-profit research institute, whose legal status under Ontario's health information privacy law allows it to collect and analyze health care and demographic data, without consent, for health system evaluation and improvement. Maternal-newborn pairs were identified in the ICES derived MOMBABY database, which captures 98% of all births in Ontario. Datasets are shown in eTable 1.

Study Design, Settings, and Participants

This population-based cohort study used linked administrative health data from Ontario, Canada, where permanent residents are eligible for universal, publicly funded healthcare.

Included were all females with two or more consecutive singleton hospital-based births at 20 to 42 weeks' gestation, between April 1, 2002 and March 31, 2022. Among mothers with more than two births in the study period, an earlier livebirth or stillbirth was randomly selected and assigned as the *first birth* in the cohort. The subsequent livebirth was assigned as the *second birth*. The cohort was restricted to women living in an income quintile (Q) Q2, 3, 4, or 5 (highest) neighbourhood at the time of the first birth, aged 15 to 50 years, and who had a valid Ontario Health Insurance Plan (OHIP) number (eTable 2).

Excluded were mothers living in a lowest income Q1 neighbourhood at the first birth, since they could not descend to a lower income Q. We have previously evaluated adverse birth outcomes among newborns of mothers living in lowest income Q1 neighbourhoods.[1,2]

The use of deidentified data in this project was authorized under section 45 of Ontario's *Personal Health Information Protection Act* and exempt from a research ethics board review. This study followed the STROBE reporting guideline.

Study Exposures

The study exposure, degree of downward neighbourhood income mobility between the first and second births, was categorized as, downward movement by one Q, two Q, or three or more Q, each relative to no downward neighbourhood income mobility

© 2025 Jairam JA et al. *JAMA Pediatrics*.

(the referent), the latter defined as residing in the same income Q at both births. For completeness, upward neighbourhood income mobility between births was also evaluated, being by one, two or three Q.

Neighbourhood income Q is an area-level measure of socioeconomic position that can change over a person's life course.[3] In this study, it is defined as the average income per single-person equivalent in a dissemination area, adjusted for household size, and derived from census area-level income data.[4,5] A mother's six-character residential postal code at the time of her first and second birth hospitalizations, were used to determine her neighbourhood income Q at each birth. This method is detailed in a previous study.[1] A postal code reflects is the smallest unit of census geography, called a dissemination area (herein, referred to as a "neighbourhood"), containing between 400 to 700 people,[6] and follows natural urban boundaries and census divisions.[7]

Study Outcomes

The primary outcome was a composite of severe neonatal morbidity or all-cause neonatal mortality (SNM-M). SNM-M was measured using a Canadian validated version of the Neonatal Adverse Outcomes Indicator (NAOI),[8] comprised of 22 components (eTable 2) – 15 neonatal complications (e.g., seizures) recorded using *International Statistical Classification of Diseases and Related Health Problems, Tenth Revision, Canada (ICD-10-CA)* diagnostic codes,[9] and 7 interventions (e.g., resuscitation by intubation and/or chest compressions) recorded using the *Canadian Classification of Health Interventions (CCI)*.[10] This indicator captures newborns who experience a severe complication during birth or the neonatal period, and it is associated with a higher risk of neonatal mortality, neonatal intensive care unit admission and extended hospital stay.[8]

The current study defined SNM-M as the presence of 1 or more NAOI components arising in the newborn's index birth admission for the second birth. If the birth hospitalization length of stay was ≤ 27 days, then SNM-M was further assessed within any subsequent rehospitalization, up to 27 days after birth. If the birth hospital duration was > 27 days, then SNM-M was assessed any time during that hospitalization, even if it surpassed 27 days.

A secondary outcome was a composite of SNM-M or stillbirth for the second pregnancy. Stillbirth was defined as a fetal death arising *in utero*, or a newborn with no

© 2025 Jairam JA et al. *JAMA Pediatrics*.

signs of life at birth, at ≥ 20 weeks' gestation.

All study variables are otherwise detailed in the online Supplement eTable 1.

Statistical Analysis

Mean values and proportions were assessed using standardized differences, contrasting mothers who experienced any downward income mobility vs. those who did not, and mothers who experienced any upward income mobility vs. those who did not, with a standardized difference greater than 0.10 indicating an important difference.[11]

For each binary outcome, modified Poisson regression generated relative risks (RR) and 95% CI, comparing within one model mothers by degree of downward neighbourhood income mobility vs. no neighbourhood income mobility, and mothers by degree of upward neighbourhood income mobility vs. no neighbourhood income mobility.[12]

Models were adjusted for maternal neighbourhood income Q (Q2, Q3, Q4, or Q5) at the first birth hospitalization; SNM-M or stillbirth at the first birth hospitalization; interpregnancy birth interval between the first and second births (6-17, 18-60, or ≥ 61 months); immigrant status (immigrant or non-immigrant); number of comorbidities within 1 to 365 days before the second birth hospitalization, measured using the Johns Hopkins Adjusted Clinical Group (ACG)® System (version 10 software), excluding pregnancy-related diagnoses, and categorized into Aggregated Diagnosis Groups (≤ 2, ≥3);[13] maternal age (16-24, 25-29, or ≥ 30 years), livebirth parity (≥ 2 or 1), and residence (rural or urban) at the second birth hospitalization. The model assessing the main outcome of SNM-M was also adjusted for any infant congenital or chromosomal anomaly identified within the second birth hospitalization.

Data were analyzed from June to November 2024, using SAS version 9.4 (SAS Institute). The magnitude of RR and precision of corresponding 95% CI indicated significant differences between exposure groups.

© 2025 Jairam JA et al. *JAMA Pediatrics*.

eReferences

1. Jairam JA, Vigod SN, Siddiqi A, et al. Neighborhood Income Mobility and Risk of Neonatal and Maternal Morbidity. *JAMA Netw Open.* 2023;6(5):e2315301.
2. Jairam JA VS, Siddiqi A, Guan J, Boblitz A, Wang X, O'Campo P, Ray JG. Morbidity and mortality of newborns born to immigrant and nonimmigrant females residing in low-income neighbourhoods. *CMAJ.* 2023;195 (15):E537-E547.
3. Shavers VL. Measurement of socioeconomic status in health disparities research. *Journal of the National Medical Association.* 2007;99(9):1013-1023.
4. Canadian Institute for Health Information. *Trends in Income-Related Health Inequalities in Canada: Methodology Notes.* Ottawa, ON: CIHI;2015.
5. Statistics Canada. Postal Code^OM Conversion File Plus (PCCF+) Version 8A, Reference Guide. December 2022 Postal codes. https://guides.library.queensu.ca/ld.php?content_id=34898339. Accessed June 15, 2024.
6. Statistics Canada. Dissemination Area. In. Ottawa, ON: Statistics Canada; 2021.
7. Buajitti E, Rosella LC. Neighbourhood socioeconomic improvement, residential mobility and premature death: a population-based cohort study and inverse probability of treatment weighting analysis. *International journal of epidemiology.* 2022.
8. Nelson CR, Ray JG, Auger N, et al. Neonatal Adverse Outcomes Among Hospital Livebirths in Canada: A National Retrospective Study. *Neonatology.* 2024.
9. Canadian Institute for Health Information. *Final report. The Canadian enhancement of ICD-10 (International Statistical Classification of Diseases and Related Health Problems, tenth revision).* Ottawa: Canadian Institute for Health Information;2001.

© 2025 Jairam JA et al. *JAMA Pediatrics*.

10. Canadian Institute for Health Information. *International Statistical Classification of Diseases and Related Health Problems, tenth revision. CCI/ICD-10.* Ottawa Canadian Institute for Health Information;2010.
11. Austin PC. Using the standardized difference to compare the prevalence of a binary variable between two groups in observational research. *Commun Stat Simul Comput.* 2009;38(6):1228-1234.
12. Zou G. A modified poisson regression approach to prospective studies with binary data. *Am J Epidemiol.* 2004;159(7):702-706.
13. John Hopkins. ACG System version 10.0 Technical Reference Guide. https://www.hopkinsacg.org/document/acg-system-version-10-0-technical-reference-guide/. Accessed May 22, 2024.

© 2025 Jairam JA et al. *JAMA Pediatrics*.
