# Supplementary material for: Maternal Downward Neighborhood Income Mobility and Ensuing Severe Neonatal Morbidity
Source: JAMA Pediatr. 2025 Feb 17;179(4):463–5. doi: 10.1001/jamapediatrics.2024.6667 (PMC11833651; doi:10.1001/jamapediatrics.2024.6667)
Supplement: Supplement 2. — Data Sharing Statement [file jamapediatr-e246667-s002.pdf]

Data Sharing Statement

Jairam. Maternal Downward Neighborhood Income Mobility and Ensuing Severe Neonatal Morbidity. *JAMA Pediatr*. Published February 17, 2025. doi:10.1001/jamapediatrics.2024.6667

Data
Data available: No
